# The Chicken *HDAC4* Promoter and Its Regulation by MYC and HIF1A

**DOI:** 10.3390/genes15121518

**Published:** 2024-11-26

**Authors:** Yingjie Wang, Ruihong Kong, Ke Xie, Xu Wang, Han Wu, Yani Zhang

**Affiliations:** 1 Jiangsu Key Laboratory of Sericultural and Animal Biotechnology, School of Biotechnology, Jiangsu University of Science and Technology, Zhenjiang 212100, China; yjwang@just.edu.cn (Y.W.);; 2 Key Laboratory of Silkworm and Mulberry Genetic Improvement, Ministry of Agriculture and Rural Affairs, Sericultural Scientific Research Center, Chinese Academy of Agricultural Sciences, Zhenjiang 212100, China; 3 Jiangsu Province Key Laboratory of Animal Breeding and Molecular Design, College of Animal Science and Technology, Yangzhou University, Yangzhou 225009, China

**Keywords:** *HDAC4*, chicken, HIF1A, transcriptional regulation

## Abstract

Background: Histone deacetylase 4 (*HDAC4*) is a member of the class II histone deacetylase family, whose members play a crucial role in various biological processes. An in-depth investigation of the transcriptional characteristics of chicken *HDAC4* can provide fundamental insights into its function. Methods: We examined *HDAC4* expression in chicken embryonic stem cells (ESC) and spermatogonial stem cells (SSC) and cloned a 444 bp fragment from upstream of the chicken *HDAC4* transcription start site. Subsequently, we constructed pEGFP-HDAC4 and a series of 5′-deletion luciferase reporter constructs, which we transfected into DF-1 cells to measure their transcriptional activity. The regulatory mechanisms of chicken *HDAC4* expression were investigated by performing trichostatin A (TSA) treatment, deleting putative transcription factor binding sites, and altering transcription factor expression levels. Results: *HDAC4* exhibited higher expression in SSC than in ESC. We confirmed that the upstream region from −295 bp to 0 bp is the core transcriptional region of *HDAC4*. TSA effectively inhibited *HDAC4* transcription, and bioinformatics analysis indicated that the chicken core *HDAC4* promoter sequence exhibits high homology with those of other avian species. The myelocytomatosis viral oncogene homolog (MYC) and hypoxia-inducible factor 1 α (HIF1A) transcription factors were predicted to bind to this core region. Treatment with TSA for 24 h resulted in the upregulation of MYC and HIF1A, which repressed *HDAC4* transcription. Conclusions: Our results provide a basis for subsequent investigations into the regulation of *HDAC4* expression and biological function.

## 1. Introduction

Gene transcription is stringently regulated by histone acetylation [1], a process that plays a critical role in spermatogenesis, oogenesis, and gametogenesis [2]. Histone deacetylase 4 (HDAC4) is a pivotal enzyme that decreases histone acetylation levels at target gene promoters [3,4], thereby influencing various biological processes. *HDAC4* expression differs between the sperm of patients with oligospermia and that of normospermic individuals [5]. Furthermore, *HDAC4*, together with *CTNNB1*, regulates the fate of spermatogonial progenitor cells [6]. *HDAC4* expression levels have also been detected in spermatogonial stem cells (SSC) by polymerase chain reaction (PCR) [7] and RNA-seq [8], suggesting that *HDAC4* may play an important role in the formation of chicken SSC. However, the function and regulation of the chicken *HDAC4* gene remains poorly understood. Consequently, investigating the promoter and regulators of chicken *HDAC4* could provide important information on its functional and regulatory mechanisms.

To date, the regulation of *HDAC4* has been predominantly studied in humans and mice, with a focus on its miRNA-mediated regulation [9,10,11] and phosphorylation [12,13] and N6-methyladenosine (m6A) modifications [14]. Furthermore, upstream stimulatory factor 1 (USF1) is shown to activate p38 mitogen-activated protein kinase (MAPK) signaling by modulating *HDAC4* promoter activity [15]. Additionally, Krüppel-like factor 7 (KLF7) and specificity protein-1 (SP1) transcription factors have been reported to bind to the *HDAC4* promoter region to increase its expression [16,17]. In avian species, dim light (6 lux) at night increases *HDAC4* expression in Indian house crows [18]. Additionally, miRNA-1 targets *HDAC4*, promoting the differentiation of duck myoblasts [19], whereas miR-365 directly suppresses *HDAC4* protein expression in primary chicken chondrocytes [20]. Despite these findings, research on the promoter of chicken *HDAC4* and its primary regulatory elements has been limited. Trichostatin A (TSA), a broad-spectrum deacetylase inhibitor, interacts efficiently with HDAC4 [21] and inhibits *HDAC4* transcription [22], although the mechanism remains ill-defined.

In this study, we cloned a 444 bp fragment from upstream of the chicken *HDAC4* transcription start site and substituted the *CMV* promoter region of the pEGFP-N1 vector with this fragment. We then generated 5′-deletion luciferase reporter constructs, transfected them into DF-1 cells, and assessed their promoter activities. To investigate how the *HDAC4* expression is regulated, we treated cells with TSA, deleted putative transcription factor binding sites, and altered transcription factor levels. Our study provides a more comprehensive understanding of the function and regulatory mechanisms of the chicken *HDAC4* gene.

## 2. Materials and Methods

### 2.1. Materials

Fertilized eggs were provided by the Institute of Poultry Science of the Chinese Academy of Agriculture Sciences for this study. The isolation of chicken embryonic stem cells (ESC) and spermatogonial stem cells (SSC) was performed according to the procedures described by Zhang et al. [8]. DF-1 cells, pEGFP-N1, pEGFP-N1-linker [23], pRL-SV40, PGL3-Basic, OE-MYC (*MYC* overexpression vector), OE-HIF1A (*HIF1A* overexpression vector), OE-NC (negative control of *MYC* and *HIF1A* overexpression vectors), KD-MYC (*MYC* knockdown vector), KD-HIF1A (*HIF1A* knockdown vector), and KD-NC (negative control of *MYC* and *HIF1A* knockdown vectors) were provided by our laboratory.

### 2.2. Bioinformatics Analysis of the HDAC4 Promoter

A number of 2000 bp upstream promoter sequences of *HDAC4* (Gene ID: NM_204313.2) were retrieved from the NCBI website and analyzed using online prediction software including BDGP’s Neural Network Promoter Prediction [24], Promoter 2.0 Prediction Server [25], FPROM [26], TSSG, TSSP, and TSSW. Additionally, potential transcription factor binding sites within the *HDAC4* core promoter region (from−295 bp to +1 bp) were predicted and analyzed using PROMO HOME PAGE [27] and JASPAR [28]. The website links for the prediction tools used are listed in Table S1.

The HDAC4 upstream sequence (from −295 bp to +1 bp) was obtained for 16 species including the chicken from the NCBI database (Table S2), and a homology analysis was conducted using MEGA7 to generate an evolutionary tree. Megalign was used to compare the HDAC4 promoter sequences of 16 species.

### 2.3. Amplification and Promoter Vectors Construction

**Figure 1:**
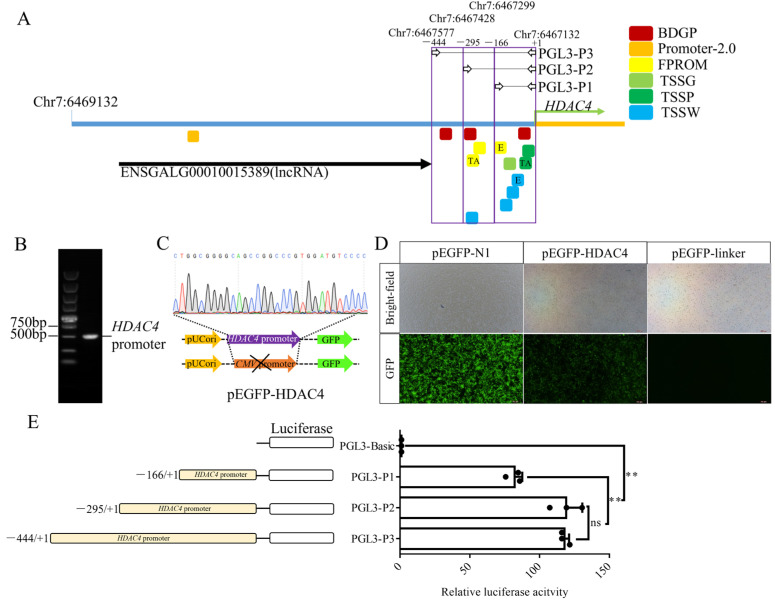
*HDAC4* promoter core region analysis. (**A**): Online prediction of the *HDAC4* promoter region. Different color boxes correspond to the promoter positions predicted by different prediction software. TA means the predicted TATA box. E means the predicted enhancer. (**B**): Full-length amplification of the *HDAC4* promoter (including PEGFP-N1 homologous arms). (**C**): Schematic diagram and sequencing chromatogram of the pEGFP-HDAC4 vector construction. (**D**): Validation of pEGFP-HDAC4 activity, DF-1 cells expressing green fluorescence. (**E**): The activity of each vector was measured by a dual-luciferase assay. Note: PGL3-P1: Fragment 1 (−166 bp to +1 bp, chr7:6467299-6467132) linked to the PGL3-Basic vector, PGL3-P2: Fragment 2 (−295 bp to +1 bp, chr7:6467428-6467132) linked to the PGL3-Basic vector, PGL3-P3: Fragment 3 (−444 bp to +1 bp, chr7:6467577-6467132) linked to the PGL3-Basic vector. ns: no significant difference, **: *p* < 0.01.

Based on the results of promoter analysis of the proximal 2000 bp upstream region of *HDAC4*, the pEGFP-HDAC4 and 5′-deletion luciferase reporter constructs (PGL3-P1, PGL3-P2, and PGL3-P3) were generated (Figure 1A). Site-deletion analysis was conducted to identify the role of MYC and HIF1A putative binding sites in the chicken *HDAC4* promoter using vectors bearing promoter deletions in MYC and HIF1A putative binding sites. NEBuilder (https://www.neb.com/, accessed on 29 October 2022) was used to design primers for vector construction, which were synthesized by Hangzhou Shangya Biotechnology. Each fragment was amplified using testicular DNA from 18.5-day-old Rugao yellow chicken embryos as a template. Table S3 lists the sequences of all primers used.

pEGFP-N1 was digested with *Ase*I (New England Biolabs, Beverly, MA, USA) and *Bam*HI (New England Biolabs, Beverly, MA, USA), whereas the PGL3-Basic was digested with *Kpn*I (Takara, Tokyo, Japan) and *Hind*III (Takara, Tokyo, Japan). The ClonExpress Ultra One Step Cloning Kit (Vazyme Biotech Co., Ltd., Shanghai, China) was used to insert different fragments containing the *HDAC4* promoter sequence into the corresponding linearized vector.

### 2.4. Cell Culture and Treatments

DF-1 cells were maintained in DMEM (Gibco, NY, USA) supplemented with 10% fetal bovine serum (FBS) (Gemini, Calabasas, CA, USA) (10% FBS-DMEM). A 12-well plate was seeded with 5 × 10^5^ cells per well. The medium was replaced after 24 h, and cells were cultured in 10% FBS-DMEM supplemented with trichostatin A (TSA) (MedChemExpress, NJ, USA) at concentrations of 10^−5^ M, 10^−6^ M, and 10^−7^ M. A control group was cultured in 10% FBS-DMEM without TSA. Cells were collected after 24 h and 48 h for qRT-PCR.

DF-1 cells were seeded in a 12-well plate at a density of 5 × 10^5^ cells per well, and transfection experiments were performed according to instructions provided with the Exfect^®^ Transfection Reagent (Vazyme Biotech Co., Ltd., Shanghai, China) at a cell density of 60%. The ratio of V_Exfect_/m_plasmid_ (in μL/μg) was 3:1. Cells were collected after 48 h for qRT-PCR.

DF-1 cells were seeded in a 24-well plate at a density of 1 × 10^5^ cells per well, and transfections were performed at a cell density of 60%. The PGL3 recombinant vector (500 ng) and pRL-SV40 (14 ng) were co-transfected following the manufacturer’s protocol for the Exfect^®^ Transfection Reagent. Additionally, a negative control group was set up by co-transfecting PGL3-Basic with pRL-SV40. Cells were collected after 48 h for the dual-luciferase assay.

The effect of transcription factors on the *HDAC4* promoter was assessed by co-transfecting PGL3 -P2 (500 ng), transcription factor overexpression/knockdown vector (500 ng), and pRL-SV40 (14 ng) into DF-1 cells. A negative control group was also established by co-transfecting PGL3-P2, OE-NC (negative control of *MYC* and *HIF1A* overexpression vectors) or KD-NC (negative control of *MYC* and *HIF1A* knockdown vectors), and pRL-SV40. Each transfection was performed in triplicate. The experiments were repeated three times using different cell batches. Cells were collected after 48 h for the dual-luciferase assay. At least three replicates were performed in all of the above experiments.

### 2.5. Quantitative Real-Time PCR (qRT-PCR)

Total RNA was extracted from test samples with RNAiso Plus (Takara, Tokyo, Japan) according to the manufacturer’s instructions. Subsequently, reverse transcription of 1 μg of RNA was performed using the PrimeScript™ RT Reagent Kit with gDNA Eraser (Takara, Tokyo, Japan). Relative quantification of the target genes was conducted using NovoStart R SYBR qPCR SuperMix Plus (Novoprotein, Shanghai, China). The reverse-transcribed cDNA was diluted 5-fold. The qRT-PCR reaction system consisted of 10 µL of 2 × NovoStart SYBR qPCR mix, 0.4 µL of each gene-specific forward and reverse primer (10 µM) (Table 1), 2 µL of cDNA, and 7.2 µL of RNase-free H_2_O. The running program was as follows: 95 °C for 1 min (initial denaturation), 95 °C for 20 s, 60 °C for 20 s, and 72 °C for 30 s, for a total of 40 cycles. *ACTB* was used as an endogenous control. The data were analyzed using a 2^−ΔΔCt^ analytical technique. qRT-PCR was performed in triplicate.

### 2.6. Dual-Luciferase Assay

The relative luciferase activity was measured using the Dual Luciferase Reporter Assay Kit (Vazyme Biotech Co., Ltd., Shanghai, China) according to the manufacturer’s instructions. After 48 h of transfection, the original medium was discarded, the cells were rinsed with PBS once, and 100 μL of 1× cell lysis buffer was added to each well. The cells were then lysed by shaking the plates for 5 min at room temperature. After centrifugation at 11,200 rpm for 2 min at room temperature, the cell lysates were transferred to 1.5 mL centrifuge tubes. Then, 10 μL of the supernatant was transferred to a new centrifuge tube and mixed with 50 μL of pre-equilibrated luciferase substrate at room temperature. The mixture was immediately used to measure firefly luciferase reporter gene activity using the Promega Glomax chemiluminescence detector. Subsequently, 50 μL of freshly prepared Renilla substrate working solution was added to the reaction mixture, followed by immediate mixing. Renilla luciferase reporter gene activity was then measured using the Promega fluorescence detector.

### 2.7. CCK8 Analysis

DF-1 cells were seeded into 96-well plates at a density of 2 × 10^4^ cells per well. After 24 h of culture, they were incubated with different concentrations of TSA/CoCl_2_ working solution. The effects of different concentrations of TSA on cells were assessed at 24 h and 48 h, whereas the effects of CoCl_2_ were assessed only at 24 h. Before the assay, the original medium was removed and replaced with 100 µL of normal medium per well. Then, 10 µL of CCK8 solution (Biosharp, Hefei, China) was added to each well. After 4 h of incubation at 37 °C, the absorbance at 450 nm was measured using a microplate reader. The absorbance values of each group were recorded for data analysis. Cell viability was calculated as (experimental well − blank well)/(control well − blank well) × 100%. Experimental wells were treated with different concentrations of TSA/CoCl_2_, control wells were untreated, and blank wells contained only medium without cells. 

### 2.8. Statistical Analysis

All data are presented as the mean ± standard errors and were processed using the SPSS 19.0 statistical software. An independent sample *t*-test was deemed suitable for comparisons between two groups, whereas one-way analysis of variance and LSD test were chosen for comparisons among multiple groups. It was defined as significant at *p* < 0.05 and extreme significance at *p* < 0.01. GraphPad Prism 6 was used to generate data histograms. 

## 3. Results

### 3.1. Deletion Analysis of the Upstream Region of the HDAC4 Gene

We found that *HDAC4* expression was significantly higher in chicken spermatogonial stem cells (SSC) than in embryonic stem cells (ESC) (Figure S1, Table S4). To obtain a better understanding of the transcriptional regulation of the chicken *HDAC4*, we analyzed the 2000 bp sequence upstream of the *HDAC4* transcription start site from NCBI and found an lncRNA (ENSGALG00010015389, Chr7:6468929-6467578) in the upstream −2000 to −444 bp region (Figure 1A). Additional analysis of this 2000 bp sequence using online prediction software such as BDGP revealed that the 444 bp sequence contained promoter elements such as a TATA box and an enhancer (Figure 1A). The 444 bp sequence was cloned (Figure 1B) and used to replace the *CMV* promoter of pEGFP-N1 to construct the pEGFP-HDAC4 (Figure 1C). We used pEGFP-N1-linker, which lacked a *CMV* promoter, as a negative control. After transfecting DF-1 cells with these vectors for 48 h, we observed that cells transfected with pEGFP-N1-linker did not exhibit fluorescence, whereas those transfected with pEGFP-HDAC4 exhibited weaker fluorescence than those transfected with pEGFP-N1 (Figure 1D).

To further characterize the *HDAC4* core promoter region, we generated a series of 5′-deletion luciferase reporter constructs, which were designed based on promoter prediction (Figure 1A). Luciferase activity was measured in transfected DF-1 cells 48 h after transfection. The results showed that cells transfected with PGL3-P1, -P2, or -P3 exhibited strong luciferase activity, with those transfected with PGL3-P2 or -P3 showing similar activity. Cells transfected with PGL3-P2 or -P3 had significantly higher activity than those transfected with PGL3-P1. This indicates the presence of important elements within the −166 to +1 bp and −295 to −166 bp regions.

### 3.2. Effect of TSA on HDAC4 Transcription

**Figure 2:**
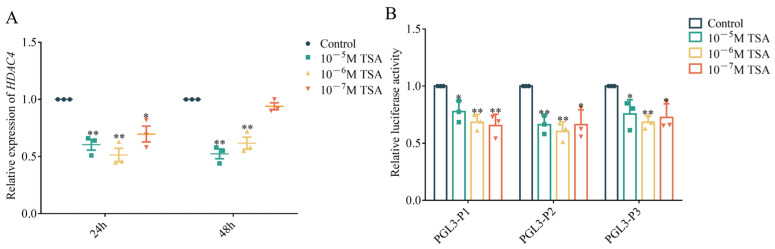
TSA inhibited *HDAC4* transcription. (**A**): qRT-PCR analysis of the expression of *HDAC4* in DF-1 after TSA treatment with different concentrations for 24 h and 48 h. (**B**): The activity of each vector was measured by a dual-luciferase assay after TSA treatment with different concentrations for 24 h. *: *p* < 0.05, **: *p* < 0.01.

To further investigate *HDAC4*’s transcriptional regulatory factors, we treated DF-1 cells with trichostatin A (TSA) and examined changes in DF-1 proliferation and *HDAC4* expression. The results showed that TSA significantly inhibited DF-1 proliferation (Figure S2). Compared to the control group, *HDAC4* was significantly inhibited after treatment with 10^−5^ M and 10^−6^ M TSA for 24 h and 48 h. While treatment with 10^−7^ M TSA for 24 h also led to significant downregulation of *HDAC4*, no significant downregulation was observed after 48 h of treatment (Figure 2A). Additionally, we treated cells transfected with luciferase reporter constructs bearing 5′ deletions of the *HDAC4* promoter with different concentrations of TSA for 24 h. The results showed that TSA significantly inhibited the relative luciferase activity of all vectors (Figure 2B). 

### 3.3. Bioinformatics of the HDAC4 Core Promoter Suggests Conserved Regulatory Sites

**Figure 3:**
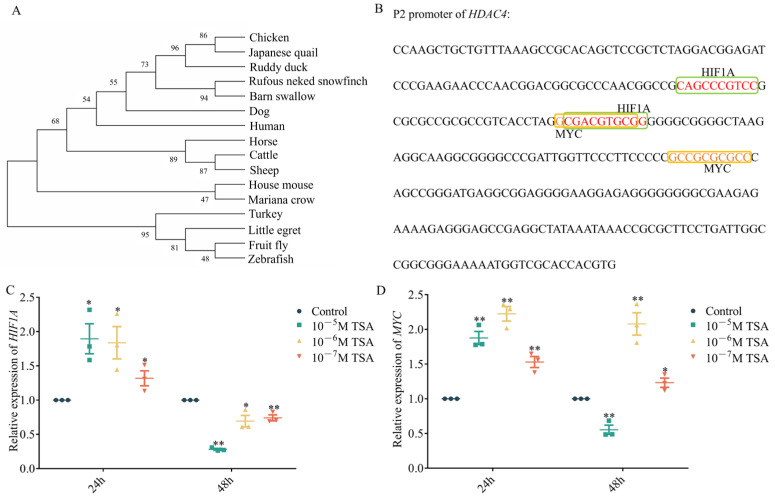
Bioinformatics analysis of the *HDAC4* core promoter region. (**A**): Homology analysis of the upstream −295 bp to +1 bp region of *HDAC4* transcription start site in different species. (**B**): Prediction of transcription factors putative binding to the upstream −295 bp to +1 bp region of chicken *HDAC4* transcription start site. (**C**,**D**): qRT-PCR analysis of the expression of *HIF1A* and *MYC* in DF-1 after TSA treatment with different concentrations for 24 h and 48 h. *: *p* < 0.05, **: *p* < 0.01.

To further explore which transcription factors regulate *HDAC4*, we first conducted a homology analysis of the −295 bp to +1 bp region upstream of the *HDAC4* transcription start site of different species. The results showed that the *HDAC4* promoter sequence in chickens is most closely related to that of Japanese quails, followed by ruddy ducks, whereas it is more distantly related to those of mammals such as humans and dogs (Figure 3A). 

Next, we identified potential binding sites for MYC and HIF1A in this region using PROMO HOME PAGE and JASPAR (Figure 3B). We also found that among the other 15 species, only the fruit fly sequence did not contain MYC and HIF1A putative binding sites (Table S2). Sequence alignment of this *HDAC4* promoter region from the 16 species showed that the MYC and HIF1A putative binding sites are highly conserved in Japanese quails, ruddy ducks, and chickens (Figure S3). Additionally, MYC and HIF1A were differentially expressed in chicken ESC and SSC (Figure S4, Table S4). We also found that both *MYC* and *HIF1A* were significantly upregulated after 24 h of TSA treatment. However, after 48 h of treatment, *HIF1A* was downregulated, whereas *MYC* was upregulated upon treatment with 10^−6^ M and 10^−7^ M TSA (Figure 3C,D).

### 3.4. Effect of MYC and HIF1A Putative Binding Sites on HDAC4 Promoter Activity

**Figure 4:**
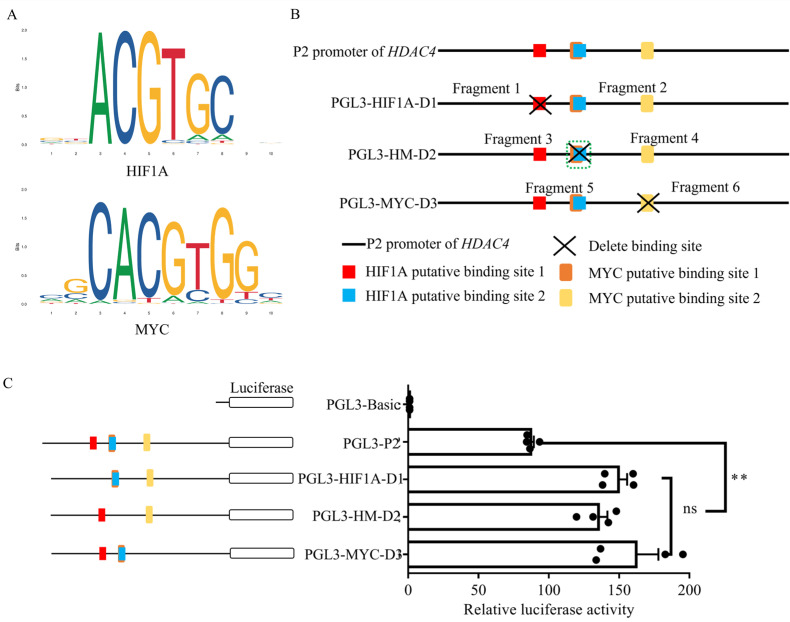
Effect of HIF1A and MYC putative binding sites on *HDAC4* promoter activity. (**A**): Sequence of transcription factor putative binding sites of HIF1A and MYC. (**B**): Schematic diagram of deletion vectors for the putative binding sites of HIF1A and MYC. (**C**): The activity of each MYC/HIF1A putative binding site deletion vector was measured by a dual-luciferase assay. ns: no significant difference, **: *p* < 0.01.

To further validate the impact of MYC and HIF1A putative binding sites on the *HDAC4* promoter, we generated recombinant vectors with deletion of MYC and HIF1A putative binding sites separately (Figure 4A,B). We then transfected them into DF-1 cells and collected samples 48 h later to measure luciferase activity. The results showed that the activity of each vector bearing a deletion in either the MYC or HIF1A putative binding site was significantly upregulated compared to PGL3-P2 (Figure 4C).

### 3.5. MYC and HIF1A Inhibit HDAC4 Transcription

**Figure 5:**
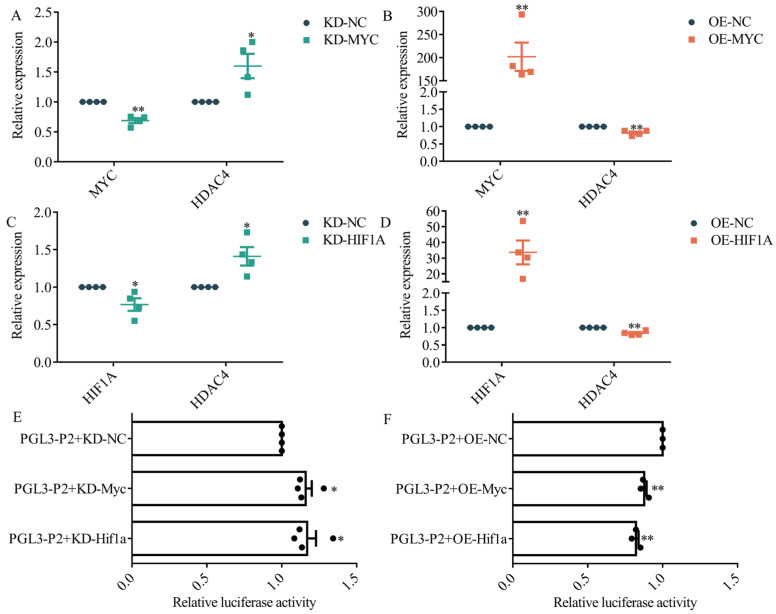
HIF1A and MYC inhibit *HDAC4* transcription. (**A**,**B**): qRT-PCR analysis of the expression of *MYC* and *HDAC4* in DF-1 after transfection with KD-MYC (*MYC* knockdown vector) or OE-MYC (*MYC* overexpression vector). (**C**,**D**): qRT-PCR analysis of the expression of *HIF1A* and *HDAC4* in DF-1 after transfection with KD-HIF1A (*HIF1A* knockdown vector) or OE-HIF1A (*HIF1A* overexpression vector). (**E**): The activity of PGL3-P2 was measured by dual-luciferase assay after transfection with KD-MYC or KD-HIF1A. (**F**): The luciferase activity of PGL3-P2 was measured by a dual-luciferase assay after transfection with OE-MYC or OE-HIF1A. *: *p* < 0.05, **: *p* < 0.01.

To determine how *HDAC4* is transcriptionally regulated by MYC and HIF1A, we separately transfected knockdown and overexpression vectors for *MYC* and *HIF1A*. After 48 h, cells were harvested, the total RNA was extracted, and qRT-PCR was performed after reverse transcription. The results showed that knockdown of either *MYC* or *HIF1A* significantly upregulated *HDAC4* expression (Figure 5A,C). Conversely, overexpression of *MYC* and *HIF1A* significantly downregulated *HDAC4* expression (Figure 5B,D).

Additionally, we co-transfected PGL3-P2 with knockdown or overexpression vectors of *MYC* and *HIF1A* into DF-1 cells. PGL3-P2 luciferase activity was significantly upregulated upon knockdown *MYC* and *HIF1A* (Figure 5E). Conversely, overexpression of *MYC* and *HIF1A* resulted in the inhibition of PGL3-P2 luciferase activity (Figure 5F).

HIF1A is a hypoxia-inducible factor, so we established a hypoxia model using CoCl_2_ [29] and found that CoCl_2_ inhibited DF-1 cell proliferation. At a concentration of CoCl_2_ of >0.4 mM, cell viability was <50% (Figure S5A). Subsequently, 0.4 mM CoCl_2_ was used to treat DF-1 cells. After 24 h, *HIF1A* and *HDAC4* were significantly up- and downregulated, respectively (Figure S5B). In addition, treatment of PGL3-P2-transfected cells with 0.4 mM CoCl_2_ significantly inhibited their luciferase activity (Figure S5C).

## 4. Discussion

Here, we observed that *HDAC4* was significantly higher in chicken spermatogonial stem cells (SSC) than in embryonic stem cells (ESC) and identified the core region of the chicken *HDAC4* promoter. Treatment with trichostatin A (TSA) for 24 h inhibited *HDAC4* expression while it increased *MYC* and *HIF1A* expression. Further investigation demonstrated that *MYC* and *HIF1A* inhibit transcription. These findings establish a foundation for future research on the role and regulatory mechanisms of *HDAC4* expression underlying the formation of chicken SSC. 

*HDAC4* is involved in numerous biological processes, with its expression being modulated by a diverse array of factors. For instance, miR-145-3p has been shown to activate autophagy in multiple myeloma cells by direct targeting HDAC4 [9]. Similarly, miR-140-5p targeting *HDAC4* decreased HDAC4 in chondrocytes [10], and miR-22 also inhibited HDAC4 expression [11]. Additionally, AMP-activated protein kinase (AMPK) phosphorylates HDAC4 [12,13], whereas the m6A reader YTH N6-methyladenosine RNA binding protein 2 (YTHDF2) recognizes m6A methylation, increasing HDAC4 stability [14]. In this study, we investigated the factors influencing HDAC4 transcription in chickens.

Given the critical regulatory function of promoter sequences for eukaryotic gene expression, research has focused on these sequences to elucidate gene function [30,31]. Typically, the promoter is situated upstream of the transcription start site at the 5’ end of the gene and can extend up to 2000 bp upstream from the start site. In this study, we analyzed the 2000 bp upstream sequence of the chicken *HDAC4* transcription start site and found another lncRNA (ENSGALG00010015389) located between −2000 bp and −444 bp. Predictive software analysis confirmed that the majority of the key promoter elements were situated between −444 bp and 0 bp. Subsequent fluorescence results of pEGFP-HDAC4 further confirmed that this region has promoter activity. Based on the transcription direction of *HDAC4* and ENSGALG00010015389, the promoter region was inferred to be a unidirectional promoter. Furthermore, truncation experiments identified that the *HDAC4* core promoter region is located between −295 bp and 0 bp.

TSA, a naturally occurring dienohydroxamic acid compound, facilitates histone acetylation, modulating cellular functions through epigenetic modifications [32]. Other studies have demonstrated that certain hydroxamic acid derivatives, such as PROTACs 9 [33] and PROTACs 11 [34], can inhibit specific HDACs, albeit not HDAC4. TSA is a broad-spectrum histone deacetylase inhibitor (HDACi) that inhibits class I and II HDACs owing to its more complex molecular structure and additional functional groups. Previous studies have demonstrated that TSA can suppress *HDAC4* expression [22,35]. Similarly, we observed that treatment with 10^−6^ M TSA resulted in the inhibition of *HDAC4* transcription. 

Transcription factors are essential trans-regulatory elements that regulate gene transcription. In this study, we predicted that transcription factors such as MYC, HIF1A, USF1, and SP1 can bind to the core promoter region of *HDAC4* (−295 bp to 0 bp). Based on the differential transcriptome analysis of chicken ESC and SSC (Table S5) [8], we initially identified transcription factors MYC and HIF1A. *MYC* is integral to the self-renewal and proliferation of various stem cell types [36,37], including SSC [38,39,40]. Our research revealed that the expression of *MYC* and *HDAC4* differed between ESC and SSC. Furthermore, compound 7c inhibits *HDAC4* expression while modulating *MYC* expression [41]. In murine cardiac tissue, Dickkopf 3 upregulates *MYC* and downregulates *HDAC4* expression [42]. These findings suggest a potential regulatory interaction between *MYC* and *HDAC4*. In this study, we observed that MYC bound to the core promoter region of *HDAC4* and repressed its transcription. This observation implies that during the formation of chicken SSC, *MYC* exerts its function via *HDAC4*. *HIF1A* is a critical regulator involved in hypoxia sensing and the maintenance of cellular oxygen homeostasis. It is notably expressed in murine spermatogonial cells [43], and its deficiency impairs the proliferation of SSC [44]. Qian et al. detected an interaction between HDAC4 and HIF1A using co-immunoprecipitation assays [45]. We observed that HIF1A represses *HDAC4* transcription. Furthermore, CoCl_2_-mediated hypoxia resulted in the inhibition of *HDAC4* promoter activity and expression, thereby reinforcing the potential relationship between *HIF1A* and *HDAC4*. These findings could be valuable for future investigations into the role of the *HIF1A*/*HDAC4* regulatory axis in chicken SSC. 

## 5. Conclusions

This study successfully located the core promoter region of chicken *HDAC4* between −295 bp to 0 bp upstream of the transcription start site. Treatment with TSA for 24 h resulted in the suppression of *HDAC4* expression while concurrently enhancing the expression of *MYC* and *HIF1A*. The *HDAC4* promoter region was found to contain putative binding sites for MYC and HIF1A, which inhibited *HDAC4* transcription. Collectively, these findings aid understanding of transcriptional regulation of the chicken *HDAC4* gene and provide a basis for further research into the role of HDAC4 in SSC.

## Figures and Tables

**Table 1:** Primers for qRT-PCR.

Gene	Gene ID	Primer (5′–3′)
*ACTB*	NM_205518.2	qF: ACCAACTGGGATGATATGGAGAA
		qR:TTGGCTTTGGGGTTCAGG
*HDAC4*	NM_204313.2	qF: GTTGGAGCAGCAGCGCATTC
		qR: TGGCTTAGTGGGTGGCTCCT
*MYC*	NM_001030952.2	qF: GGTCTTCCCCTACCCGCTCA
		qR:CGGACTGTGGTGGGGCTTAC
*HIF1A*	NM_001396327.1	qF: CAAGAGCAACCAACCAGCCCT
		qR: TGATCAAAGGAGCGTAGCTGGA

## Data Availability

The original contributions presented in the study are included in the article/Supplementary Materials; further inquiries can be directed to the corresponding author.

## Supplementary Materials

The following supporting information can be downloaded at: https://www.mdpi.com/article/10.3390/genes15121518/s1, Figure S1: Expression of *HDAC4* in chicken ESC and SSC. **: *p* < 0.01. Figure S2: TSA reduces the viability of DF-1 cells. Cell viability of DF-1 measured by CCK8 assay after TSA treatment with different concentrations for 24 h and 48 h. *: *p* < 0.05, **: *p* < 0.01. Figure S3: Multiple alignment of the *HDAC4* promoter from 16 species. Prediction of mostly conserved MYC and HIF1A putative binding sites in chicken. Figure S4: Expression of *MYC* and *HIF1A* in chicken ESC and SSC. **: *p* < 0.01. Figure S5: CoCl_2_ inhibits *HDAC4* promoter activity. (**A**): Cell viability of DF-1 measured by CCK8 assay after CoCl_2_ treatment with different concentrations for 24 h. (**B**): qRT-PCR analysis of the expression of *HDAC4* and *HIF1A* in DF-1 after 0.4 mM CoCl_2_ treatment for 24 h. (**C**): The activity of PGL3-P2 was measured by a dual-luciferase assay after 0.4 mM CoCl*_2_* treatment for 24 h. *: *p* < 0.05, **: *p* < 0.01. Table S1: Websites of online prediction tools. Table S2: *HDAC4* prediction promoter information and transcription factor binding motifs prediction in 16 species. Table S3: All primers used for the plasmid construction. Table S4: ΔCt and ΔΔCt of genes in chicken ESC and SSC. Table S5: RPKM of genes in chicken ESC and SSC.
