# Supplementary material for: The Chicken HDAC4 Promoter and Its Regulation by MYC and HIF1A
Source: Genes (Basel). 2024 Nov 26;15(12):1518. doi: 10.3390/genes15121518 (PMC11675110; doi:10.3390/genes15121518)
Supplement: Supplementary file 1 [file genes-15-01518-s001.zip › genes-3245622-supplementary.pdf]

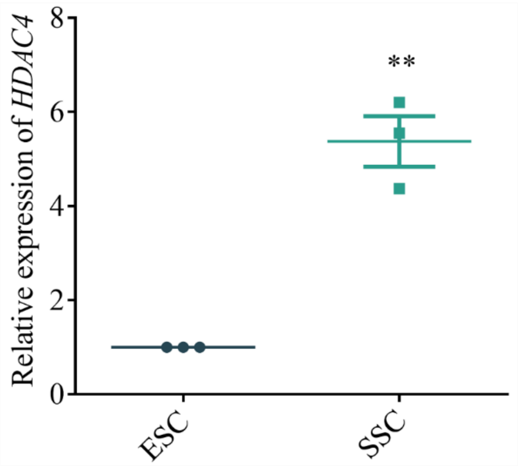

Figure S1: Expression of *HDAC4* in chicken ESC and SSC. **: p < 0.01.

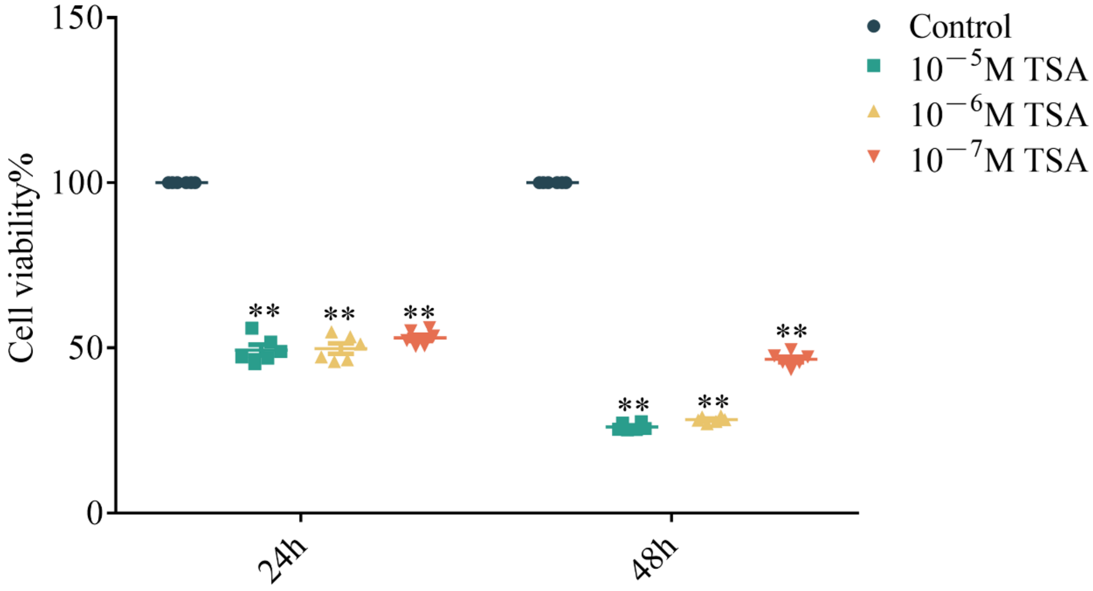

Figure S2: TSA reduces the viability of DF-1 cells. Cell viability of DF-1 measured by CCK8 assay after TSA treatment with different concentrations for 24 h and 48 h. *: $p < 0.05$, **: $p < 0.01$.

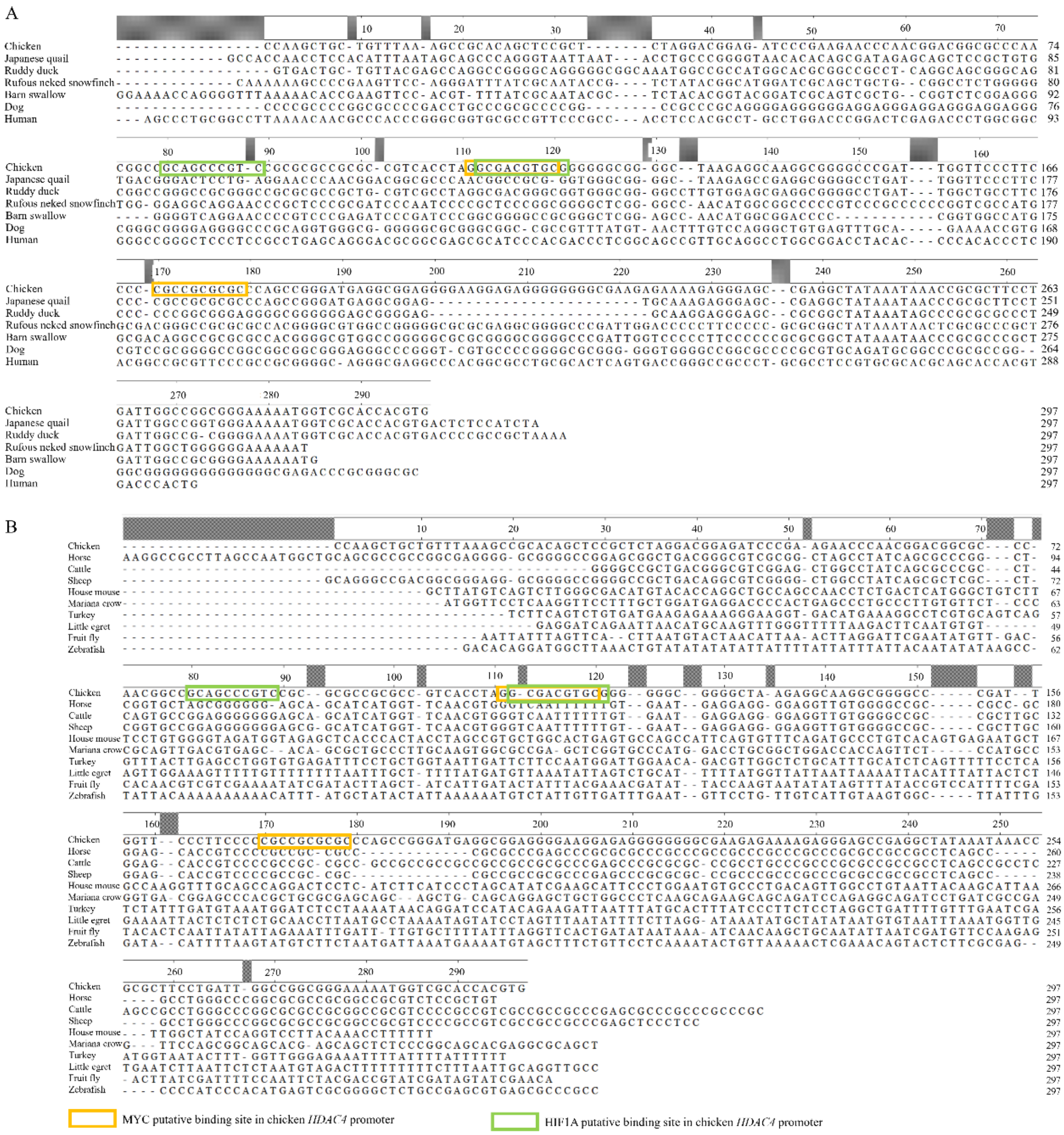

Figure S3: Multiple alignment of the *HDAC4* promoter from 16 species. Prediction of mostly conserved MYC and HIF1A putative binding sites in chicken.

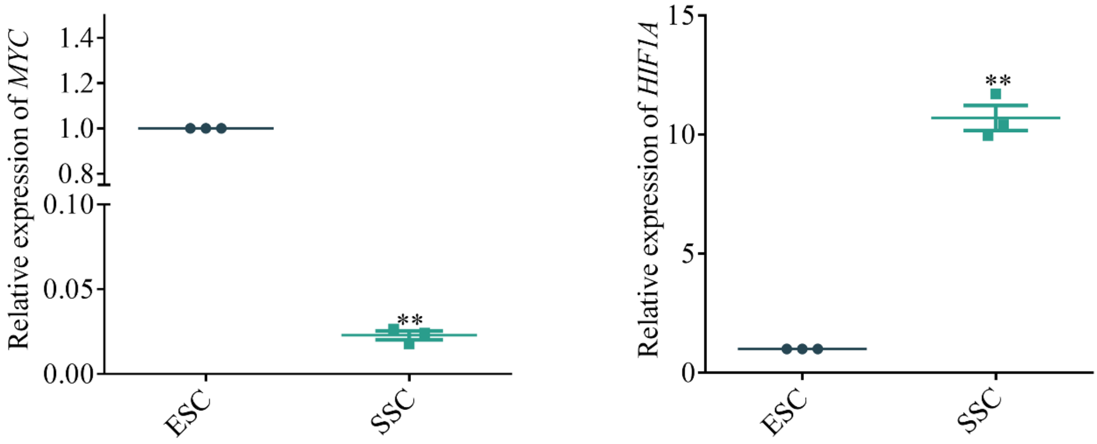

Figure S4: Expression of *MYC* and *HIF1A* in chicken ESC and SSC. **: $p < 0.01$.

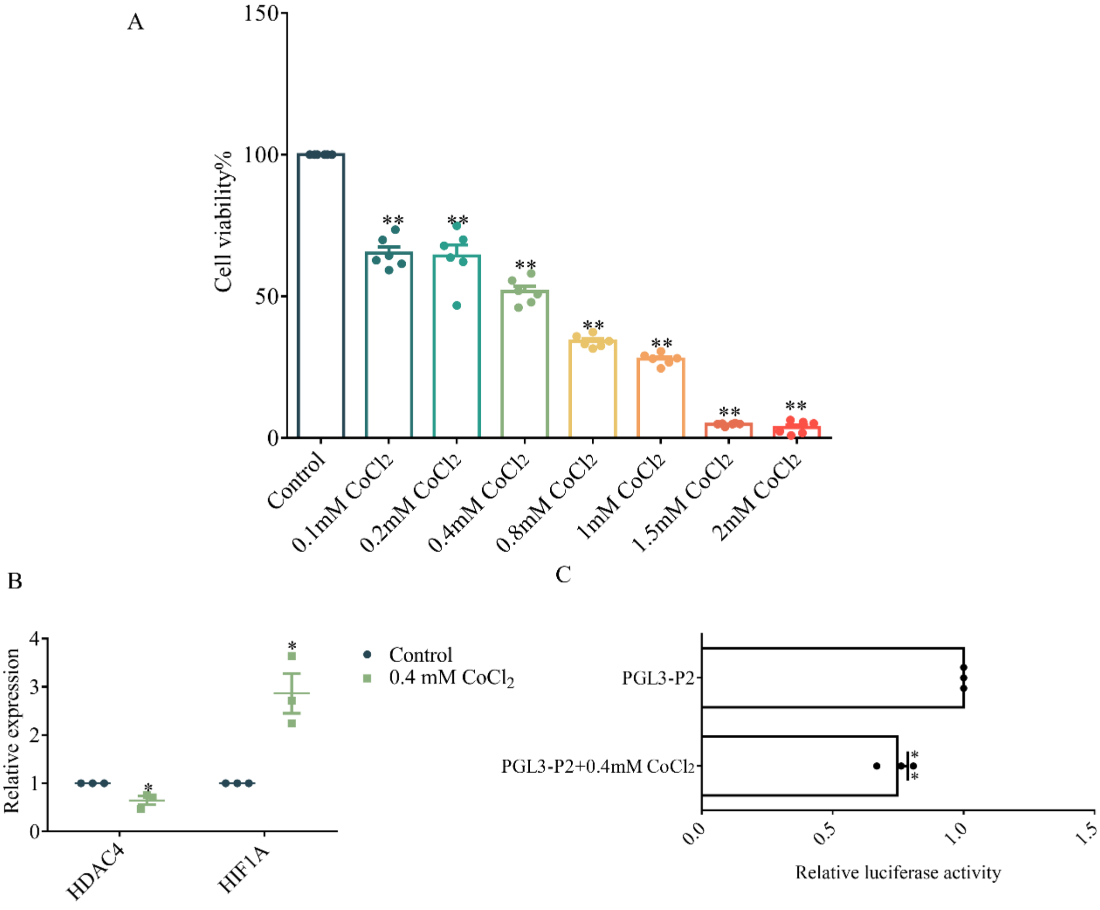

Figure S5: $CoCl_2$ inhibits *HDAC4* promoter activity. A: Cell viability of DF-1 measured by CCK8 assay after $CoCl_2$ treatment with different concentrations for 24 h. B: qRT-PCR analysis of the expression of *HDAC4* and *HIF1A* in DF-1 after 0.4 mM $CoCl_2$ treatment for 24 h. C: The activity of PGL3-P2 was measured by a dual-luciferase assay after 0.4 mM $CoCl_2$ treatment for 24 h. *: $p < 0.05$, **: $p < 0.01$.

Table S1 Website of online prediction tool

Name of online forecasting software	Website
NCBI	https://www.ncbi.nlm.nih.gov/
BDGP	http://www.fruitfly.org/seq_tools/promoter.html
Promoter 2.0	http://www.cbs.dtu.dk/servic-es/Promoter/
FPROM	http://www.softberry.com/berry.phtml?topic=fprom&group=programs&subgroup=promoter
TSSG	http://www.softberry.com/berry.phtml?topic=tssg&group=programs&subgroup=promoter
TSSP	http://www.softberry.com/berry.phtml?topic=tssp&group=programs&subgroup=promoter
TSSW	http://www.softberry.com/berry.phtml?topic=tssw&group=programs&subgroup=promoter
PROMO HOME PAGE	https://alggen.lsi.upc.es/cgi-bin/promo_v3/promo/promoinit.cgi?dirDB=TF_8.3
JASPAR	https://jaspar.elixir.no/

Table S2 *HDAC4* prediction promoter information and transcription factor binding motifs prediction in 16 species

Species	Gene ID	Location	Transcription factor binding motifs				
			HIF1A	MYC	USF1	KLF7	SP1
Chicken	NM_204313.2	Chr7:6467428-6467132	√	√	√	√	√
Japanese quail	XM_015867781.2	Chr7:2888334-2888630	√	√	√	√	√
Ruddy duck	XM_035330833.1	Chr7:17370875-17371171	√	√	√	√	√
Rufous neked snowfinch	XM_041465775.1	Chr:177149-177445	√	√	√	√	√
Barn swallow	XM_040068885.1	Chr7:352204543-352204839	√	√	√	√	√
Dog	XM_038435961.1	Chr25:49976665-49976961	√	√	√	√	√
Human	NM_001378414.1	Chr2:239401649-239401945	√	√	√	√	√
Horse	XM_023642722.1	Chr6:25130016-25130312	√	√	√	√	√
Cattle	XM_024990281.1	Chr3:118512913-118513209	√	√	√	√	√
Sheep	XM_042244201.1	Chr1:2120622-2120918	√	√	√	√	√
House mouse	NM_207225.2	Chr1:92123424-92123720	√	√	×	×	√
Mariana crow	XM_042035230.1	Chr:518151-517855	√	√	√	√	√
Turkey	XM_010713207.3	Chr7:907249-907545	√	√	√	×	√
Little egret	XM_035890270.1	Chr:548440-548736	√	√	×	×	×
Fruit fly	NM_001272578.2	ChrX:13285632-13285928	×	×	×	×	×
Zebrafish	NM_001039358.2	Chr9:46276409-46276705	√	√	√	√	√

Note: √ means that there are corresponding transcription factor motifs for the sequence. × means that there is no corresponding transcription factor motif for the sequence.

Table S3 All primers used for the plasmid construction

Name of fragment	Length of product	Primer sequence（5'-3'）
pEGFP-HDAC4	495bp	F: taccgccatgcattagttat*taat*TTCCCGCGATAAGCAATG
		R: caccatggtggcgaccggtgg*atcc*CACGTGGTGCGACCATTTTTC
PGL3-P1	214bp	F1: atttctctatcgataggtac*c*GGCTAAGAGGCAAGGCGG
		R1: ccaacagtaccggaatgcca*agctt*CACGTGGTGCGACCATTTTTC
PGL3-P2	343bp	F2: atttctctatcgataggtac*c*CCAAGCTGCTGTTTAAAGCC
		R1: ccaacagtaccggaatgcca*agctt*CACGTGGTGCGACCATTTTTC
PGL3-P3	492bp	F3: atttctctatcgataggtac*c*TTCCCGCGATAAGCAATG
		R1: ccaacagtaccggaatgcca*agctt*CACGTGGTGCGACCATTTTTC
Fragment 1	111bp	F2: atttctctatcgataggtac*c*CCAAGCTGCTGTTTAAAGCC
		dR1: cgcggcgcgcCGGCCGTTGGGCGCCGTC
Fragment 2	242bp	dF2: ccaacggccgGCGCGCCGCGCCGTCACC
		R1: ccaacagtaccggaatgcca*agctt*CACGTGGTGCGACCATTTTTC
Fragment 3	142bp	F2: atttctctatcgataggtac*c*CCAAGCTGCTGTTTAAAGCC
		dR3: gccccgccccCTAGGTGACGGCGCGGCG
Fragment 4	210bp	dF4: cgtcacctagGGGGCGGGGCTAAGAGGC
		R1: ccaacagtaccggaatgcca*agctt*CACGTGGTGCGACCATTTTTC
Fragment 5	200bp	F2: atttctctatcgataggtac*c*CCAAGCTGCTGTTTAAAGCC
		dR5: atcccggctgGGGGGAAGGGAACCAATC
Fragment 6	410bp	dF6: cccttccccCAGCCGGGATGAGGCGGA
		R1: ccaacagtaccggaatgcca*agctt*CACGTGGTGCGACCATTTTTC

Note: The lowercase letters are homologous arms.

Table S4 △Ct and △△Ct of genes in chicken ESC and SSC

GENE	△Ct /△△Ct	ESC1	ESC2	ESC3	SSC1	SSC2	SSC3
HDAC4	△Ct	9.44	9.31	9.36	6.97	7.18	6.73
	△△Ct	0	0	0	-2.47	-2.13	-2.63
MYC	△Ct	3.95	3.78	4.07	9.32	9.60	9.30
	△△Ct	0	0	0	5.38	5.81	5.24
HIF1A	△Ct	8.48	8.57	8.23	4.93	5.19	4.91
	△△Ct	0	0	0	-3.55	-3.38	-3.32

Table S5 RPKM of genes in chicken ESC and SSC

GENE	ESC	SSC
HDAC4	4.402040847	11.34832483
MYC	114.2623131	53.82539343
HIF1A	27.83692873	55.31587762
USF1	93.78298735	62.44712252
SP1	26.47718284	33.67928695

Table S

Species	HIF1A	MYC	USF1	KLF7	SP1
Chicken	√	√	√	√	√
Japanese quail	√	√	√	√	√
Ruddy duck	√	√	√	√	√
Rufous neked snowfinch	√	√	√	√	√
Barn swallow	√	√	√	√	√
Dog	√	√	√	√	√
Human	√	√	√	√	√
Horse	√	√	√	√	√
Cattle	√	√	√	√	√
Sheep	√	√	√	√	√
House mouse	√	√	×	×	√
Mariana crow	√	√	√	√	√
Turkey	√	√	√	×	√
Little egret	√	√	×	×	×
Fruit fly	×	×	×	×	×
Zebrafish	√	√	√	√	√
